# Birmingham COPD Cohort: a cross-sectional analysis of the factors associated with the likelihood of being in paid employment among people with COPD

**DOI:** 10.2147/COPD.S119467

**Published:** 2017-01-11

**Authors:** Kiran K Rai, Rachel E Jordan, W Stanley Siebert, Steven S Sadhra, David A Fitzmaurice, Alice J Sitch, Jon G Ayres, Peymané Adab

**Affiliations:** 1 Institute of Applied Health Research; 2 The Department of Business and Labour Economics; 3 Institute of Clinical Sciences, University of Birmingham, Edgbaston, Birmingham, UK

**Keywords:** chronic obstructive pulmonary disease, work, employed, breathlessness, severity, VGDF, UK

## Abstract

**Background:**

Employment rates among those with chronic obstructive pulmonary disease (COPD) are lower than those without COPD, but little is known about the factors that affect COPD patients’ ability to work.

**Methods:**

Multivariable analysis of the Birmingham COPD Cohort Study baseline data was used to assess the associations between lifestyle, clinical, and occupational characteristics and likelihood of being in paid employment among working-age COPD patients.

**Results:**

In total, 608 of 1,889 COPD participants were of working age, of whom 248 (40.8%) were in work. Older age (60–64 years vs 30–49 years: odds ratio [OR] =0.28; 95% confidence interval [CI] =0.12–0.65), lower educational level (no formal qualification vs degree/higher level: OR =0.43; 95% CI =0.19–0.97), poorer prognostic score (highest vs lowest quartile of modified body mass index, airflow obstruction, dyspnea, and exercise (BODE) score: OR =0.10; 95% CI =0.03–0.33), and history of high occupational exposure to vapors, gases, dusts, or fumes (VGDF; high VGDF vs no VGDF exposure: OR =0.32; 95% CI =0.12–0.85) were associated with a lower probability of being employed. Only the degree of breathlessness of BODE was significantly associated with employment.

**Conclusion:**

This is the first study to comprehensively assess the characteristics associated with employment in a community sample of people with COPD. Future interventions should focus on managing breathlessness and reducing occupational exposures to VGDF to improve the work capability among those with COPD.

## Introduction

Chronic obstructive pulmonary disease (COPD) is a progressive lung disease characterized by airflow obstruction.^1^ It affects 6%–10% of the global population^2^ and is an important cause of morbidity and mortality worldwide.^3^,^4^ In 2003, an estimated 44% of the UK COPD population were of working age, of whom around one-quarter were not in work due to their COPD.^5^ These estimates are higher in the USA, where 69.0% of the people with COPD are of working age,^6^ and more than one-third are not working due to their COPD.^7^ Furthermore, there is consistent evidence across a range of observational studies^8^–^13^ and a review^14^ that patients with COPD have lower employment rates than those without COPD, and they are also less likely to be in work compared to people with asthma or those with other chronic diseases.^8^ Likewise, there is some evidence to suggest that when out of work, COPD patients are less likely to reenter the labor market compared to those with no chronic conditions.^8^ Estimates from US data suggest that COPD-attributed work loss costs the US economy ≥18.5 billion annually.^14^

There is increasing evidence that being in work is good for physical and mental health and well-being.^15^ There are also economic advantages for both the individual and society. Thus, interventions to support people with chronic diseases to remain in or return to work are recommended.^16^,^17^ However, a better understanding of the disease-related aspects that affect employment is needed to inform the planning of such interventions.

Few studies have examined the factors associated with employment among patients with COPD, and although a range of behavioral and disease-related factors are implicated, the findings are inconsistent. Although current smoking, severity of airflow obstruction, degree of symptoms, and the number and type of comorbidities have been shown to be associated with lower employment rates,^11^,^18^,^19^ other studies have not confirmed this.^13^,^20^ Occupational exposures may also contribute to the lower employment rates among those with COPD,^19^ although evidence for this is also limited and conflicting.^19^,^20^ Furthermore, methodological weakness, including lack of adjustment for important confounders and use of self-report as a measure of COPD status, limits interpretation. In addition, most of the previous studies have been based in the USA, Nordic countries, and the Netherlands, which have different social and welfare settings compared to the UK, potentially impacting the motivation to remain in work. Therefore, in this study, baseline data from the Birmingham COPD Cohort Study in the UK were used to assess the factors associated with employment among people with COPD in the community.

## Methods

### Study design and participants

Participants with COPD from the Birmingham COPD Cohort Study were included in the present study.^21^ Patients on primary care COPD registers and those who were newly diagnosed with COPD through a case-finding trial^22^ were recruited between June 2012 and July 2014, from 71 general practices within the West Midlands, UK. This analysis included only participants with COPD who were of working age (<65 years). All participants provided written informed consent to take part in the Birmingham COPD Cohort Study.

### Ethical approval

The Birmingham COPD Cohort Study is part of The Birmingham Lung Improvement StudieS (BLISS). As this was a cohort study, ethical approval was required from a national ethics committee. The Birmingham COPD Cohort received ethical approval from the National Research Ethics Service Committee West Midlands – Solihull (Reference: 11/WM/0304).

### Measures

As a part of the baseline assessment, all participants were asked to complete detailed questionnaires and underwent a range of clinical assessments. All these assessments were undertaken by trained research assistants (RAs), using standardized protocols and validated instruments.

#### Sociodemographic characteristics and lifestyle

Validated questionnaires were used to collect information on age, sex, smoking status, highest educational level achieved, and gross household income. Social deprivation was measured by using the Index of Multiple Deprivation (IMD) 2010 score^23^ based on individual participant postcodes.

#### Clinical characteristics

Relevant questionnaire items included breathlessness (the Medical Research Council [MRC] score),^24^ COPD specific quality of life measure (COPD Assessment Test [CAT]),^25^ exacerbations (self-reported steroid or antibiotic treatment for an exacerbation in the previous 12 months), and comorbidities (self-reported physician diagnosis of cardiovascular disease, diabetes, gastrointestinal disease, cancer, depression, osteoarthritis, rheumatoid arthritis, hay fever, eczema, and allergies). Height (to the nearest 0.1 cm) and weight (to the nearest 0.1 kg) measurements were obtained and used to derive body mass index (BMI). Pre- and postbronchodilator spirometries, according to the American Thoracic Society/European Respiratory Society 2005 guidelines,^26^ were carried out by the RAs who received intense training based on a modified version of the Association for Respiratory Technology and Physiology spirometry course. The severity of airflow obstruction was determined by using postbronchodilator spirometry; % predicted forced expiratory volume in 1 s (FEV_1_; postbronchodilator) was calculated using Global Lung Function Initiative reference equations^27^ (% of the predicted values for the patients’ age, height, sex, and ethnicity, with reference to a healthy nonsmoking population), and Global Initiative for Chronic Obstructive Lung Disease (GOLD) staging criteria^28^ were used to measure the severity of airflow obstruction. The sit-to-stand test (counting the number of times the participant stood from a seated position, without using their arms to stand up) was used to assess exercise capacity (carried out postbronchodilator).^29^

A multicomponent prognostic score (modified body mass index, airflow obstruction, dyspnea, and exercise [BODE] index) was calculated using the sit-to-stand test as a proxy for the 6-min walk test (6MWT) by using cutoff points derived from a study that assessed the correlation between the two measures.^29^

#### Occupational characteristics

Irrespective of the current employment status of the participants who were included in the present study, trained RAs obtained detailed information about their occupational history through face-to-face interviews by using a validated questionnaire from the Work Employment Relations Study.^30^ The trained RAs used this information to manually generate a 4-digit standard occupational classification (SOC) 2010 code for the longest held occupation by using the Computer-Assisted Structured Coding Tool (CASCOT) software.^31^

For accurate and standardized SOC coding, one of the authors (KKR) delivered a standardized training program to all the RAs. The training program provided instructions on software navigation, the factors to consider when coding occupations (job title and job tasks), and practice sessions for coding a range of occupations. RAs were encouraged to contact KKR in the event of ambiguity (difficult to code occupations) for advice. A random sample of 25–30 participants who were assessed by each RA was also interviewed by KKR, who independently generated the SOC code, and was blind to the code originally generated by the RA, for quality control. The RA’s code was overwritten when there were any discrepancies, and further training offered to the RA if required.

A job exposure matrix (JEM) derived from the airborne chemical exposure (ACE) JEM^32^ was used to assign the risk (ie, none, low, medium, and high) of occupational exposures to vapors, gases, dusts, or fumes (VGDF) for each SOC code. This was developed based on previous published work^32^ (through personal communication between SSS and KKR). Self-reported employment type (employed for wages/self-employed) was collected for a subset of participants.

### Primary outcome

The primary outcome was employment. The participants were categorized as being employed if they reported any current paid employment (full-time or part-time) or as not employed if they reported no paid employment at the baseline visit.

### Statistical analyses

All the analyses were conducted by using STATA version 13.0. Multivariable logistic regression models were used to examine the associations between paid employment and sociodemographic, lifestyle, clinical, and occupational characteristics.

Univariate models were fitted with each characteristic separately, followed by the final multivariable model. Known clinically important covariates (ie, age, sex, smoking status, and number of comorbidities), clinical severity, and socioeconomic status were included in the multivariable model. The exact measures of clinical severity (ie, MRC score, CAT score, number of exacerbations, and modified BODE index) and socioeconomic status (ie, education level, income, and IMD score) used in the multivariable model were chosen by using the likelihood ratio test, based on which covariate significantly contributed to the model (likelihood ratio *P*<0.05).

An additional model was fitted to further explore the importance of individual components of the BODE index, adjusting for age, sex, smoking status, education level, number of comorbidities, exposures to VGDF, and the other components of the modified BODE index.

In a subset of participants for whom data were available, the effect of employment type (employed/self-employed) on being in work was also assessed.

## Results

### Characteristics of the participants

**Figure 1:**
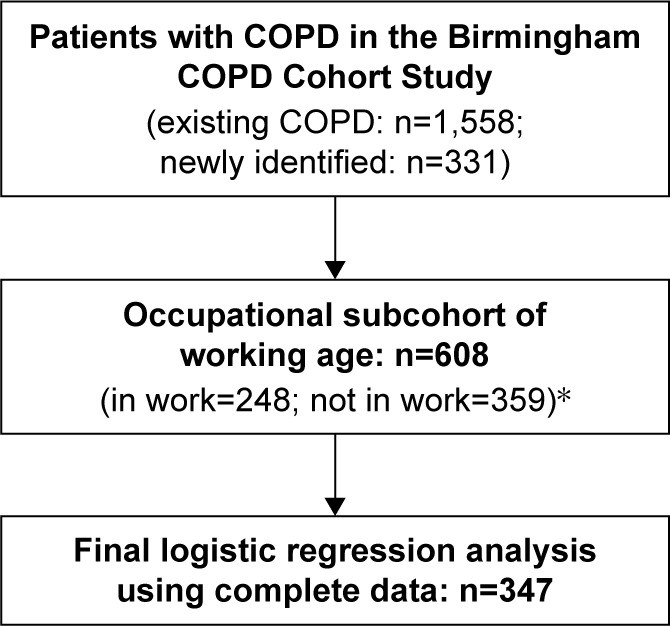
Participant flow chart for study participants from the Birmingham COPD Cohort Study. **Note:** *Employment status data missing for one participant at the baseline visit.

Among 1,889 participants with COPD in the Birmingham COPD Cohort Study, there were 608 (32.2%) participants who were of working age and form the group that is studied, of whom 248 (40.8%) participants reported being in work (Figure 1).

Table 1 summarizes the characteristics of study participants. Around half (n=287; 47.2%) of the participants were aged 60–64 years, 278 (49.7%) participants were current smokers, 248 (51.5%) participants had no formal educational qualification, and 321 (54.3%) participants had a manual occupational background (ie, skilled trade, process/plant/machine operatives, elementary). More than one-third of the participants were either obese or severely obese (n=205; 35.3%), and the majority had at least one comorbidity (n=519; 85.3%). The main comorbidities included: cardiovascular disease (n=268; 49.2%), depression (n=181; 32.9%), and gastrointestinal disease (n=160; 27.7%). There was a range of severity of disease described by dyspnea, CAT score, modified BODE score, and airflow obstruction. One-third of the participants (n=197; 33.1%) had either medium or high occupational exposures to VGDF.

Although there were some differences in the characteristics between men and women, the most notable were the differences in types of occupation. Men were also more likely to experience higher workplace exposures to VGDF in their longest held occupation.

### Factors associated with the likelihood of being in paid employment

In the univariate analyses, participants who were female, older, current smokers, had a lower level of education, and a higher deprivation score were less likely to be in paid employment (although not all the differences were statistically significant). In addition, participants with cardiovascular disease, diabetes, gastrointestinal disease, osteoarthritis, rheumatoid arthritis, and a greater number of comorbidities were less likely to be in work.

A greater number of exacerbations, more severe breathlessness, higher CAT score, greater airflow obstruction, poorer prognostic score, and a history of higher VGDF exposure were also associated with lower probability of being in paid employment.

In the multivariable model, age, education level, modified BODE index, and occupational exposures to VGDF remained independently associated with employment status (Table 2). Women were less likely to be in work compared with men, although this did not reach statistical significance.

### Relationship between components of the BODE index and likelihood of being in paid employment

Table 3 shows the relative effects of the different components of the BODE index. The present study found that only the dyspnea component significantly contributed to the overall model (likelihood ratio test, *P*<0.01) and was significantly independently associated with employment. In the adjusted models, the likelihood of being in paid employment was 64% lower for those with a modified MRC dyspnea score of 4, compared to those with a score of 0–1 (adjusted odds ratio [OR] =0.36; 95% confidence interval [CI] =0.15–0.85).

Point estimates of airflow obstruction and exercise capacity were suggestive of possible trends, but were not statistically significant. The BMI component within BODE was not associated with employment.

### Effect of employment type

Data on employment type were available from a subset of participants (n=140; 23.0%). After adjustment, participants with a self-employed occupational background were more likely to be in work compared to those who were employed for wages (OR =3.84; 95% CI =0.55–26.88), although the CIs were wide.

## Discussion

### Key results

This is the first study in the UK to explore the association between sociodemographic, clinical, and occupational characteristics and the likelihood of being in paid employment among people with COPD. This study found that only 40% of working-age COPD participants in the present cohort were in work, and the factors independently associated with the reduced probability of being in work were being older, having a lower educational level, worse breathlessness, and high occupational exposures to VGDF.

### Findings in relation to other studies

Compared with a recent UK labor force survey among a working-age population (16–64 years), the present COPD study sample had lower employment rates than the UK general population (employment rate 73.5%), those with a long-term health condition (59.6%), and those with a disability (46.1%).^33^ Although the age and sex structures of the populations may differ and partly account for these differences, the findings are consistent with a previous work.^8^

The finding from the present study that older age and lower levels of education were independently associated with reduced probability of employment is consistent with the findings of other published research among patients with COPD,^13^ and these are the well-recognized factors affecting employment in the general population.^34^,^35^ Recent UK employment trends show that while there has been a rise in the proportion of women and a fall in the proportion of men who are now in work, employment rates among women remain lower than those among men.^36^ Smoking status has also been shown to be associated with employment status in the UK; those who are unemployed are nearly twice more likely to smoke.^37^ Although not statistically significant, the present findings suggested trends in the same direction for sex and smoking and may not have been clearly demonstrated due to the limited sample size.

It was surprising to find that the presence of multiple comorbidities was not independently associated with the likelihood of employment; especially as in the univariate analyses, it appeared to be an important factor. Although this finding is consistent with those reported in other studies on patients with COPD,^13^,^20^ the direction of the point estimates and width of the CIs suggest that multiple comorbidities may be an important factor affecting employment, which might also be clarified with a larger sample.

This seems to be the first study to use a standardized measure of exposure to VGDF (the ACE JEM^32^) within a model assessing the factors associated with employment status among COPD patients. Previous research methods have used self-report^19^ and professional judgment^20^ as measures of determining these workplace exposures. This variation in measurement may lend some explanation for the conflicting results of the role of occupational exposures in employment among COPD patients.^19^,^20^ It is also worth noting that this study was based within the West Midlands, UK, an area that has a history of industrial occupations, where the likelihood of workplace exposures to VGDF may be higher than other areas within the UK. Although this may affect the prevalence estimates observed in the present study, the associations between high exposures to VGDF and the lower probability of being in work remain valid.

An interesting finding was that, of the several clinical factors related to disease severity, only the level of dyspnea was independently associated with employment and contributed to the final model. The other measures of disease severity within the composite BODE index – airflow obstruction and exercise capacity – were less important. However, although not statistically significant, they did indicate weaker trends, and a larger sample size may have increased the precision of the results.

Findings from the previous studies have revealed conflicting results between the association of airflow obstruction and employment status.^11^,^13^,^19^,^20^ These differences may be explained by the methodological differences between the studies as well as the measurement used to describe disease severity (eg, by using GOLD staging or mean FEV_1_ % predicted). There has been little work assessing the impact of other measures of disease severity, including the degree of breathlessness or other symptoms, on the ability to work.

Currently, it is accepted that airflow obstruction does not fully capture the impact of COPD on patients’ lives,^38^,^39^ and this study confirms that symptoms are more important in explaining whether a patient is in paid employment.

### Strengths and limitations

As this is a cross-sectional study, it was not possible to determine causality. Factors such as income, deprivation, and quality of life were significantly associated with employment in the univariate analyses. However, these are particularly susceptible to reverse causation and therefore were not included in the multivariable models. Workplace exposures to VGDF may also have preceded COPD diagnosis and may be an important factor in the development of COPD. Some data were based on self-report, including number of comorbidities and exacerbations, possibly introducing errors in prevalence rates and diluting the findings. In addition, there was a low response rate for some measures (eg, smoking status, exercise capacity, and exacerbations), which may have led to less reliable estimates. Due to low participation in the Birmingham COPD Cohort Study, the study sample may not be representative of the full range of patients with COPD in primary care. However, the associations observed remain valid. While there are a number of established and validated job exposure matrices available, the present study used the ACE JEM because it is the only matrix based on the UK occupational classification codes (SOC), allowing the data set to be easily merged and analyzed. Furthermore, it is the only JEM with a clear and detailed methodology, with estimates based on a comprehensive range of airborne occupational exposures. For the BODE prognostic index, a modified version was used – replacing the 6MWT with the sit-to-stand test as a measure of exercise capacity, and this requires further validation.

### Implications for practice and research

The findings from the present study reveal some important differences among patients with COPD who are in paid employment compared to those who are not in paid employment, suggesting that there are possible opportunities to modify certain factors to improve work capability. Breathlessness was the most important clinical factor identified. The UK National Institute for Health and Care Excellence guidelines for COPD^1^ provide guidance for health care professionals on the management of breathlessness. This may involve providing advice on better medication management (eg, the correct inhaler usage), advice on smoking cessation and referral to pulmonary rehabilitation (eg, among those who have a MRC score ≥3), or self-management advice. Therefore, health care professionals have a role to work alongside patients and focus on improving the management of their breathlessness, in particular among the working-age population. Within the workplace, patients (alone or in conjunction with their employer) may want to consider modifying aspects of their job, which may otherwise exacerbate their breathlessness, for example, carrying heavy loads, regular use of stairs, or tasks that involve lifting and bending.

The second important modifiable factor identified was high exposure to VGDF. This suggests that COPD patients should potentially undergo an occupational health (OH) assessment to determine the level of exposure to VGDF in their working environment and, thus, for OH services to advise accordingly on how those in high exposure jobs may be able to modify their job role, job tasks, or working environment.

Although being in work is associated with better health among patients with COPD, there is a need for prospective longitudinal studies to establish the temporal relationships between disease severity, occupational exposures to VGDF, and employment. Further research is also required to determine the effectiveness of managing breathlessness and reducing workplace exposures on work capability among patients with COPD.

## Conclusion

This study demonstrates the high levels of unemployment among those with COPD. Two potentially modifiable factors associated with the reduced probability of being in work were identified: greater breathlessness and high workplace exposures to VGDF. Future interventions should focus on improving the management of breathlessness and reducing workplace exposures to VGDF in order to help improve the work capability in those with COPD.

## Figures and Tables

**Table 1:** Sociodemographic, lifestyle, clinical, and occupational characteristics of COPD patients of working age recruited to the Birmingham COPD Cohort Study

Participant characteristics	Male, number (%); n=343	Female, number (%); n=265	Total number (%); n=608
**Sociodemographic characteristics**			
Age categories (years)			
38–49	40 (11.7)	28 (10.6)	68 (11.2)
50–59	125 (36.4)	128 (48.3)	253 (41.6)
60–64	178 (51.9)	109 (41.1)	287 (47.2)
Education level			
Degree or higher level	33 (12.1)	25 (12.0)	58 (12.0)
A level/AS level or equivalent	21 (7.7)	15 (7.2)	36 (7.5)
GCSE, CSE, O level, or equivalent	73 (26.7)	67 (32.1)	140 (29.1)
No formal qualification	146 (53.5)	102 (48.8)	248 (51.5)
IMD score quintiles			
$\begin{array}{l} 1–2\\ 3\\ 4–5 \end{array}\;↓\text{increasing deprivation}$	110 (32.4)	74 (27.9)	184 (30.4)
	70 (20.6)	66 (24.9)	136 (22.5)
	160 (47.1)	125 (47.2)	285 (47.1)
**Lifestyle characteristics**			
Smoking status			
Never smoked	23 (7.2)	19 (7.9)	42 (7.5)
Ex-smoker	135 (42.5)	104 (43.2)	239 (42.8)
Current smoker	160 (50.3)	118 (49.0)	278 (49.7)
**Clinical characteristics**			
MRC dyspnea grade			
$\begin{array}{l} 1\\ 2\\ 3\\ 4–5 \end{array}\;↓\text{Increasing breathlessness}$	81 (24.8)	44 (17.2)	125 (21.4)
	57 (17.4)	46 (18.0)	103 (17.8)
	68 (20.8)	62 (24.2)	130 (22.3)
	121 (37.0)	104 (40.6)	225 (38.6)
CAT score			
$\begin{array}{l} \text{Low}\;(\text{high QOL})\\ \text{Medium}\\ \text{High}\\ \text{Very high}\;(\text{poor QOL}) \end{array}\;↓\text{Increasing symptom impact}$	40 (15.1)	26 (12.5)	66 (14.0)
	102 (38.5)	72 (34.6)	174 (36.8)
	87 (32.8)	79 (38.0)	166 (35.1)
	36 (13.6)	31 (14.9)	67 (14.2)
Degree of airflow obstruction (GOLD stage)^a^,^b^			
Mild	91 (28.0)	84 (32.9)	175 (30.2)
Moderate	154 (47.4)	133 (52.2)	287 (49.5)
Severe to very severe	80 (24.6)	38 (14.9)	118 (20.3)
Exacerbations in the last 12 months			
0	146 (47.7)	82 (33.7)	228 (41.5)
1–2	110 (36.0)	92 (37.9)	202 (36.8)
3+	50 (16.3)	69 (28.4)	119 (21.7)
Modified BODE index score quartiles^a^			
$\begin{array}{l} 1\\ 2\\ 3\\ 4 \end{array}\;↓\text{Increasing severity}$	103 (41.2)	82 (39.1)	185 (40.2)
	72 (28.8)	57 (27.1)	129 (28.0)
	46 (18.4)	49 (23.3)	95 (20.7)
	29 (11.6)	22 (10.5)	51 (11.1)
Number of comorbidities			
0	64 (18.7)	25 (9.4)	89 (14.6)
1–2	140 (40.8)	110 (41.5)	250 (41.1)
3+	139 (40.5)	130 (49.1)	269 (44.2)
Comorbidities			
Cardiovascular disease	155 (51.0)	113 (46.9)	268 (49.2)
Depression	86 (28.1)	95 (38.8)	181 (32.9)
Gastrointestinal disease	89 (27.2)	71 (28.4)	160 (27.7)
Osteoarthritis	42 (14.4)	47 (19.8)	89 (16.8)
Diabetes	46 (15.3)	27 (11.2)	73 (13.5)
Rheumatoid arthritis	29 (10.0)	37 (15.5)	66 (12.5)
Cancer	18 (6.0)	25 (10.3)	43 (7.9)
BMI categories			
Underweight (<18.5)	8 (2.5)	8 (3.1)	16 (2.8)
Normal (18.5–24.9)	83 (25.5)	73 (28.4)	156 (26.8)
Overweight (25–29)	132 (40.6)	73 (28.4)	205 (35.2)
Obese (30–39.0)	96 (29.5)	76 (29.6)	172 (29.6)
Severe obesity (≥40.0)	6 (1.9)	27 (10.5)	33 (5.7)
Mean sit-to-stand repetitions (SD)	20.8 (6.6)	19.8 (6.6)	20.4 (6.6)
**Occupational characteristics**			
Occupational background (SOC 2010)			
Managers, directors, and senior officials	20 (5.9)	17 (6.6)	37 (6.2)
Professional	27 (8.0)	28 (10.9)	55 (9.2)
Associate professional and technical	28 (8.3)	11 (4.3)	39 (6.5)
Administrative and secretarial	5 (1.5)	48 (18.7)	53 (8.9)
Skilled trade	116 (34.2)	12 (4.7)	128 (21.5)
Caring, leisure, and other services	7 (2.1)	38 (14.8)	45 (7.6)
Sales and customer service	7 (2.1)	21 (8.2)	28 (4.7)
Process, plant, and machine operatives	87 (25.7)	39 (15.2)	126 (21.1)
Elementary	42 (12.4)	43 (16.7)	85 (14.3)
Exposures to VGDF in longest held job			
$\begin{array}{l} \text{None}\\ \text{Low}\\ \text{Midium}\\ \text{High} \end{array}\;↓\text{Increasing occupational airborne exposure}$	65 (19.2)	129 (50.4)	194 (32.6)
	129 (38.1)	73 (28.5)	202 (34.0)
	78 (23.0)	49 (19.1)	127 (21.3)
	67 (19.8)	5 (2.0)	72 (12.1)

**Notes:**

a Airflow obstruction based on postbronchodilator FEV_1_ % predicted (mild: FEV_1_ ≥80% predicted; moderate: 50% ≤ FEV_1_ <80% predicted; severe: 30% ≤ FEV_1_ <50% predicted, and very severe: FEV_1_ <30% predicted);

b Includes 94 participants (male: n=38 and female: n=56) who did not have airflow obstruction on the day of assessment. Total numbers may differ due to some missing data.

**Abbreviations:** BMI, body mass index; BODE, body mass index, airflow obstruction, dyspnea, and exercise; CAT, COPD Assessment Test; COPD, chronic obstructive pulmonary disease; FEV_1_, forced expiratory volume in 1 s; GOLD, Global Initiative for Chronic Obstructive Lung Disease; IMD, index of multiple deprivation; MRC, Medical Research Council; QOL, quality of life; SD, standard deviation; SOC, standard occupational classification; VGDF, vapors, gases, dusts, or fumes.

**Table 2:** Relationship between sociodemographic, lifestyle, clinical, and occupational characteristics and being in paid employment among working-age COPD patients

Characteristics	Number (%) employed n=248	Univariate model ORs (95% CI) for being employed	Final multivariable model ORs (95% CI) for being employed^a^
Sex			
Male	147 (42.9)	1.0	1.0
Female	101 (38.3)	0.83 (0.60–1.15)	0.60 (0.36–1.01)
Age categories (years)			
38–49	38 (55.9)	1.0	1.0
50–59	122 (48.4)	0.74 (0.43–1.27)	0.55 (0.24–1.26)
60–64	88 (30.7)	0.35 (0.20–0.60)	0.28 (0.12–0.65)
Education level			
Degree or higher level	40 (69.0)	1.0	1.0
A level/AS level or equivalent	21 (58.3)	0.63 (0.27–1.50)	0.65 (0.23–1.90)
GCSE, CSE, O level, or equivalent	64 (45.7)	0.38 (0.20–0.72)	0.55 (0.25–1.25)
No formal qualification	68 (27.5)	0.17 (0.09–0.32)	0.43 (0.19–0.97)
Smoking status			
Never smoked	19 (45.2)	1.0	1.0
Ex-smoker	103 (43.1)	0.92 (0.47–1.78)	0.98 (0.37–2.60)
Current smoker	106 (38.3)	0.75 (0.39–1.44)	0.79 (0.29–2.10)
Number of comorbidities			
0	44 (50.0)	1.0	1.0
1–2	118 (47.2)	0.89 (0.55–1.45)	1.14 (0.52–2.49)
3+	86 (32.0)	0.47 (0.29–0.77)	0.70 (0.32–1.57)
MRC dyspnea grade			
$\begin{array}{l} 1\\ 2\\ 3\\ 4–5 \end{array}\;↓\text{Increasing breathlessness}$	79 (63.2)	1.0	
	59 (57.3)	0.78 (0.46–1.33)	
	67 (51.5)	0.62 (0.38–1.02)	
	36 (16.0)	0.11 (0.07–0.18)	
CAT score			
$\begin{array}{l} \text{Low}\;(\text{high QOL})\\ \text{Medium}\\ \text{High}\\ \text{Very high}\;(\text{poor QOL}) \end{array}\;↓\text{Increasing symptom impact}$	42 (63.6)	1.0	
	88 (50.6)	0.58 (0.33–1.05)	
	52 (31.3)	0.26 (0.14–0.47)	
	13 (19.7)	0.14 (0.06–0.31)	
Degree of airflow obstruction			
Mild	91 (52.0)	1.0	
Moderate	111 (38.7)	0.58 (0.40–0.85)	
Severe to very severe	38 (32.2)	0.44 (0.27–0.71)	
Exacerbations in the last 12 months			
0	106 (46.5)	1.0	
1–2	91 (45.1)	0.94 (0.65–1.38)	
3+	34 (28.6)	0.46 (0.29–0.74)	
Modified BODE index score quartiles			
$\begin{array}{l} 1\\ 2\\ 3\\ 4 \end{array}\;↓\text{Increasing severity}$	116 (62.7)	1.0	1.0
	63 (48.8)	0.57 (0.36–0.90)	0.84 (0.48–1.47)
	25 (26.3)	0.21 (0.12–0.37)	0.38 (0.19–0.74)
	5 (9.8)	0.06 (0.02–0.17)	0.10 (0.03–0.33)
Exposures to VG			
$\begin{array}{l} \text{None}\\ \text{Low}\\ \text{Midium}\\ \text{High} \end{array}\;↓\text{Increasing occupational airborne exposure}$	94 (48.0)	1.0	1.0
	86 (42.6)	0.80 (0.54–1.19)	0.93 (0.51–1.71)
	47 (37.9)	0.66 (0.42–1.05)	0.91 (0.43–1.91)
	18 (24.7)	0.36 (0.19–0.65)	0.32 (0.12–0.85)

**Notes:**

a Multivariable model includes age, sex, smoking status, education level, number of comorbidities, modified BODE index, and exposures to VGDF;

b Airflow obstruction based on postbronchodilator FEV_1_ % predicted (mild: FEV_1_ ≥80% predicted; moderate: 50% ≤ FEV_1_ <80% predicted; severe: 30% ≤ FEV_1_ <50% predicted; and very severe: FEV_1_ <30% predicted);

c Includes 94 participants (male: n=38; and female: n=56) who did not have airflow obstruction on the day of assessment. Total numbers may differ due to some missing data.

**Abbreviations:** BODE, body mass index, airflow obstruction, dyspnea, and exercise; CAT, COPD Assessment Test; CI, confidence interval; COPD, chronic obstructive pulmonary disease; FEV_1_, forced expiratory volume in 1 s; GOLD, Global Initiative for Chronic Obstructive Lung Disease; MRC, Medical Research Council; OR, odds ratio; QOL, quality of life; VGDF, vapors, gases, dusts, or fumes.

**Table 3:** Relationship between the components of the modified BODE index and likelihood of being in paid employment

The BODE index components	Unadjusted OR (95% CI)	Adjusted OR^a^ (95% CI)	*P*-value
B: BMI			
>21	1.0 (reference)	1.0 (reference)	
≤21	0.83 (0.46–1.50)	0.80 (0.33–1.92)	0.61
O: Airflow obstruction (postbronchodilator FEV_1_ % predicted)			
≥65	1.0 (reference)	1.0 (reference)	*P* for trend =0.60
50–65	0.63 (0.40–0.98)	0.95 (0.47–1.89)	
35–49	0.61 (0.37–1.01)	0.83 (0.39–1.77)	
≤35	0.45 (0.21–0.95)	0.82 (0.25–2.71)	
D: Dyspnea (mMRC score)			
0–1	1.0 (reference)	1.0 (reference)	*P* for trend <0.01
2	0.69 (0.45–1.07)	1.18 (0.64–2.18)	
3	0.14 (0.08–0.26)	0.23 (0.08–0.62)	
4	0.11 (0.06–0.19)	0.36 (0.15–0.85)	
E: Exercise capacity (modified to using sit-to-stand repetitions)			
≥26	1.0 (reference)	1.0 (reference)	*P* for trend =0.09
21–25	0.71 (0.41–1.25)	0.84 (0.40–1.77)	
16–20	0.36 (0.21–0.62)	0.80 (0.38–1.71)	
≤15	0.13 (0.07–0.24)	0.41 (0.16–1.03)	

**Note:**

a Adjusted for age, sex, smoking status, education level, number of comorbidities, exposures to VGDF, and the other components of the modified BODE index.

**Abbreviations:** BMI, body mass index; BODE, body mass index, airflow obstruction, dyspnea, and exercise; CI, confidence interval; COPD, chronic obstructive pulmonary disease; FEV_1_, forced expiratory volume in 1 s; mMRC, modified Medical Research Council; OR, odds ratio; VGDF, vapors, gases, dusts, or fumes.
